# Efficacy and safety of Apatinib in stage IV sarcomas: experience of a major sarcoma center in China

**DOI:** 10.18632/oncotarget.16293

**Published:** 2017-03-16

**Authors:** Feng Li, Zhichao Liao, Jun Zhao, Gang Zhao, Xubin Li, Xiaoling Du, Yun Yang, Jilong Yang

**Affiliations:** ^1^ Department of Bone and Soft Tissue Tumor, Tianjin Medical University Cancer Institute & Hospital, Tianjin, People's Republic of China; ^2^ National Clinical Research Center of Cancer, Tianjin Medical University Cancer Institute & Hospital, Tianjin, People's Republic of China; ^3^ Department of Pathology, Tianjin Medical University Cancer Institute & Hospital, Tianjin, People's Republic of China; ^4^ Department of Radiology, Tianjin Medical University Cancer Institute & Hospital, Tianjin, People's Republic of China; ^5^ Department of Diagnostics, Tianjin Medical University, Tianjin, People's Republic of China

**Keywords:** sarcoma, Apatinib, efficacy, progression free survival, clinical benefit response

## Abstract

**Purpose:**

This study was conducted to review the efficacy and safety of Apatinib in stage IV sarcoma patients who failed previous chemotherapy.

**Materials and Methods:**

The clinical information on 16 patients with stage IV sarcomas who failed in prior chemotherapy and subsequently received Apatinib treatment was collected. Apatinib was given 500mg/daily and 4 weeks as a cycle. All patients had at least one measurable extracranial tumor according to Response Evaluation Criteria In Solid Tumors 1.0 criteria. Progression free survival (PFS), overall survival (OS), objective response rate (ORR), disease control rate (DCR) and treatment-related adverse effects (AEs) were reviewed and evaluated.

**Results:**

Patients was administered Apatinib for 0 to 9 cycles with the median of 3.2 cycles. Median follow-up time was 8.4 months (1 to 12 months). Ten of 16 patients received at least 1 complete cycle of Apatinib treatment were eligible for the efficacy analysis. The median PFS was 8.84 months. Two patients achieved partial response (PR) and 6 patients achieved stable disease (SD). Two patients were evaluated as progression disease (PD) and one patient died of disease progression. The ORR was 20.0% (2/10) and the DCR was 80.0% (8/10). The most common grade 3/4 treatment-related AEs were hypertension (18.7%), hand-foot syndrome (12.5%) and proteinuria (6.3%). No drug-related severe AEs occurred.

**Conclusion:**

CApatinib treatment in this exploratory study exhibited objective efficacy and manageable toxicity in stage IV sarcoma patients who failed in chemotherapy. This result supports future random controlled trial to further define Apatinib activity in stage IV sarcomas.

## INTRODUCTION

Sarcoma is a group of malignant tumors that is originated in mesenchymal tissue. In the United States (US), sarcoma accounts for approximately 1% of adult and 15% of pediatric malignancies [1]. Generally, sarcoma is divided into bone sarcoma and soft tissue sarcoma (STS). As the most common primary malignant bone sarcoma in children and adolescents, osteosarcoma is a typical and representative malignant mesenchymal tumor [2]. Approximately 20% of the osteosarcoma patients have metastases at the time of diagnosis, and the lung is the most common site of metastasis, followed by bone [2, 3]. STSs are a heterogeneous group of rare sarcomas with distinct clinical and pathologic characteristics and the most common type is pleomorphic undifferentiated sarcoma (25% ∼ 35%), followed by liposarcoma (25% ∼ 30%), leiomyosarcoma (12%), synovial sarcoma (10%) and malignant peripheral nerve sheath tumor (6%) [4]. In 2016, an estimated 12,310 people are diagnosed with STS in US, and approximately 4,990 would die of this disease [5]. Recently, the National Central Cancer Registry of China, composed of 72 local population-based cancer registries providing a population coverage of about 85.5 million people (6.5% of the national population), estimated that there were 28,000 new bone sarcoma cases and 20,700 bone sarcoma deaths in China in 2015 [6].

The prognosis of sarcoma patients in stage IV is poor. For STS, the response rate of chemotherapy is only 20-35% and the median survival time is about 12 months. The 5 year survival rate is lower than 10% reported in several large-scale studies [7]. Similarly, patients with advanced osteosarcoma also have a poor prognosis with the overall survival rate of 0%∼50% [8]. Although chemotherapy plays a major role in the treatment of advanced soft tissue sarcoma and bone sarcoma, the classic chemotherapy agents such as ifosfamide (IFO), doxorubicine (ADM), methotrexate (MTX), cisplatin (DDP), dacarbazine (DTIC), gemcitabine (GEM) and docetaxel (TXT) is not curative [9, 10]. Combination chemotherapy or dose-dense regimens have largely failed to improve the response rates [11, 12]. Long-term using of cytotoxic drugs increased the risk of toxicity in patients. For example, cumulative dose and dose intensity of doxorubicin caused cardiomyopathy and an associated mortality risk [13, 14]. Therefore, there is an urgent need for a new therapy for the treatment of advanced sarcomas.

Angiogenesis is a key process for tumor growth and metastasis. Thus, anti-angiogenesis is considered an important form of tumor therapy [15]. Apatinib is a small molecular inhibitor of Vascular Epithelial Growth Factor Receptor −2 (VEGFR-2). It highly selectively binds and inhibits VEGFR-2, blocks downstream signaling, prevents VEGF-mediated endothelial cell migration and proliferation, and inhibits neovascularization with potential antiangiogenic and antitumor activity [16, 17]. It is similar to PTK787/ZK222584 (Valatinib) and shown superior *in vivo* efficacy in heterologous transplantation studies than valatinib [18]. Phase I and phase II Apatinib trials have demonstrated encouraging antitumor activity and manageable toxicities in gastric cancer, mammary cancer and non-small-cell lung cancer [18–20]. Apatinib has been granted by the China Food and Drug Administration (CFDA) in 2014 for the treatment of advanced gastric cancer or adenocarcinomas of the esophagogastric junction. At our Sarcoma Center, metastatic sarcoma patients which failed in chemotherapeutic treatments have experimented with Apatinib treatment and showed response. Here, we reviewed these data and evaluated the efficacy and safety of Apatinib as a single agent in these sarcoma patients in stage IV.

## RESULTS

### Patients and treatment

**Figure 1:**
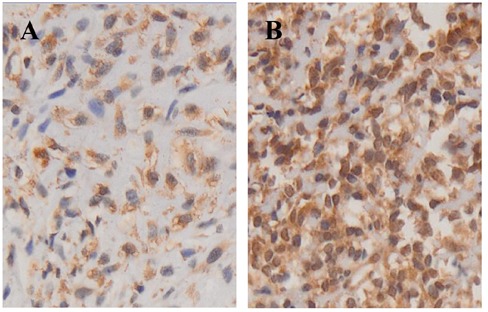
Immunohistochemical images show the different expression of VEGFR-2 in 2 osteosarcoma patients **A**. Moderate VEGFR-2 expression in patient 14 (40×). **B**. Strong VEGFR-2 expression in patient 2 (40×).

From August, 2015 to August, 2016, 16 patients in our center received Apatinib treatment (Table 1). Median age was 45 years (range 16-83 years). All patients had stage IV disease according to the American Joint Committee on Cancer (AJCC) staging and lung was the most common metastatic site of disease. The majority of the patients had received prior wide resection (87.5%), and some patients received radiotherapy (37.5%). All patients had received prior chemotherapy according to the NCCN (National Comprehensive Cancer Network) guideline. All these patients were given more than a dose of Apatinib and then the survival as well as security analysis were carried out (Table 2). We have performed immunochemistry staining of VEGFR2 in available tissues from 2 patients and detected strong to moderate expression (Figure 1).

**Table 1:** The clinical characteristics of 16 sarcoma patients in stage IV treated with Apatinib

Patient	Sex	Age	Histology	Surgery times before Apatinib	Radiotherapy before Apatinib	Chemotherapy before Apatinib	ECOG status	Initial Apatinib Dose(mg)	Cycles	Efficacy
1	Female	55	Hemangiopericytoma	2	Yes	Endostar+PTX+DTIC	1	500	0	-
2	Female	44	Osteosarcoma	1	No	DOC+CBP+THP, DOC+IFO+VP-16	1	500	11	PR
3	Female	37	Undifferentiated pleomorphic sarcoma	1	Yes	DDP+CBP+Endostar	1	500	2	PD
4	Female	43	Chondrosarcoma	1	No	EPI+IFO+DDP	1	500	0	-
5	Male	72	Rhabdomyosarcoma	4	Yes	IFO+Mesna+ADM+DTIC	1	500	0	-
6	Female	83	Liposarcoma	0	No	IFO+EPI+ L-OHP+Mesna	1	500	0	-
7	Female	37	Synovial sarcoma	1	No	PTX+EPI+L-OHP	1	500	10	SD
8	Male	22	Fibrosarcoma	1	Yes	DTIC+IFO+THP+Mesna	0	500	6	SD
9	Female	42	Leiomyosarcoma	0	No	IFO+THP+DTIC+VCR	1	500	3	PD
10	Male	55	Synovial sarcoma	4	No	DTIC+IFO+THP+Mesna	1	500	9	SD
11	Female	34	Fibrosarcoma	9	Yes	DTIC+IFO+THP+Mesna	0	500	0	-
12	Male	67	Undifferentiated pleomorphic sarcoma	1	Yes	DOC+IFO+THP	1	500	0	-
13	Female	55	Osteosarcoma	1	No	DOC+IFO+VP-16	1	500	6	SD
14	Male	16	Osteosarcoma	1	No	MTX+IFO+DDP+ADM MTX+DDP+ADM TXT+GEM	0	500	6	PR
15	Male	20	Osteosarcoma	1	No	DOC+IFO+VP-16	1	500	1	SD
16	Female	36	Fibrosarcoma	2	No	ADM+IFO	1	500	2	SD

Abbreviations: ECOG, Eastern Cooperative Oncology Group; CR, complete response; PR, partial response; SD, stable disease; PD, progression disease.

**Table 2:** Adverse events in the Apatinib treatment

Adverse event	Grade 1* (n, %)	Grade 2 (n, %)	Grade 3 (n, %)	Total
Neutropenia	2(12.5)	1(6.3)	-	3(18.8)
Thrombocytopenia	2(12.5)	-	-	2(12.5)
Bilirubin increased	2(12.5)	1(6.3)	-	3(18.8)
Transaminase increased	1(6.3)	1(6.3)	-	2(12.5)
Hematuria	1(6.3)	-	-	1(6.3)
Proteinuria	2(12.5)	4(25.0)	1(6.3)	7(43.8)
Hypertension	1(6.3)	1(6.3)	3(18.7)	5(31.3)
Hand-foot syndrome	1(6.3)	4(25.0)	2(12.5)	7(43.8)
Pain	2(12.5)	2(12.5)	-	4(25.0)
Fatigue	1(6.3)	1(6.3)	-	2(12.5)
Mucositis	-	2(12.5)	-	2(12.5)
Anorexia	-	1(6.3)	-	1(6.3)
Dizziness	1(6.3)	-	-	1(6.3)
Fever	1(6.3)	-	-	1(6.3)
Diarrhea	1(6.3)	-	-	1(6.3)

### Efficacy

Six of the 16 patients received less than one full cycle of treatment thus were not eligible for evaluation of the efficacy (Table 1). Among these 6 patients, three could not continue to take the medication after 15 days due to economic difficulties. Another 2 patients stopped treatment after 7 days due to the sudden progression of the disease. One patient had cerebral hemorrhage and required hospitalization.

**Figure 2:**
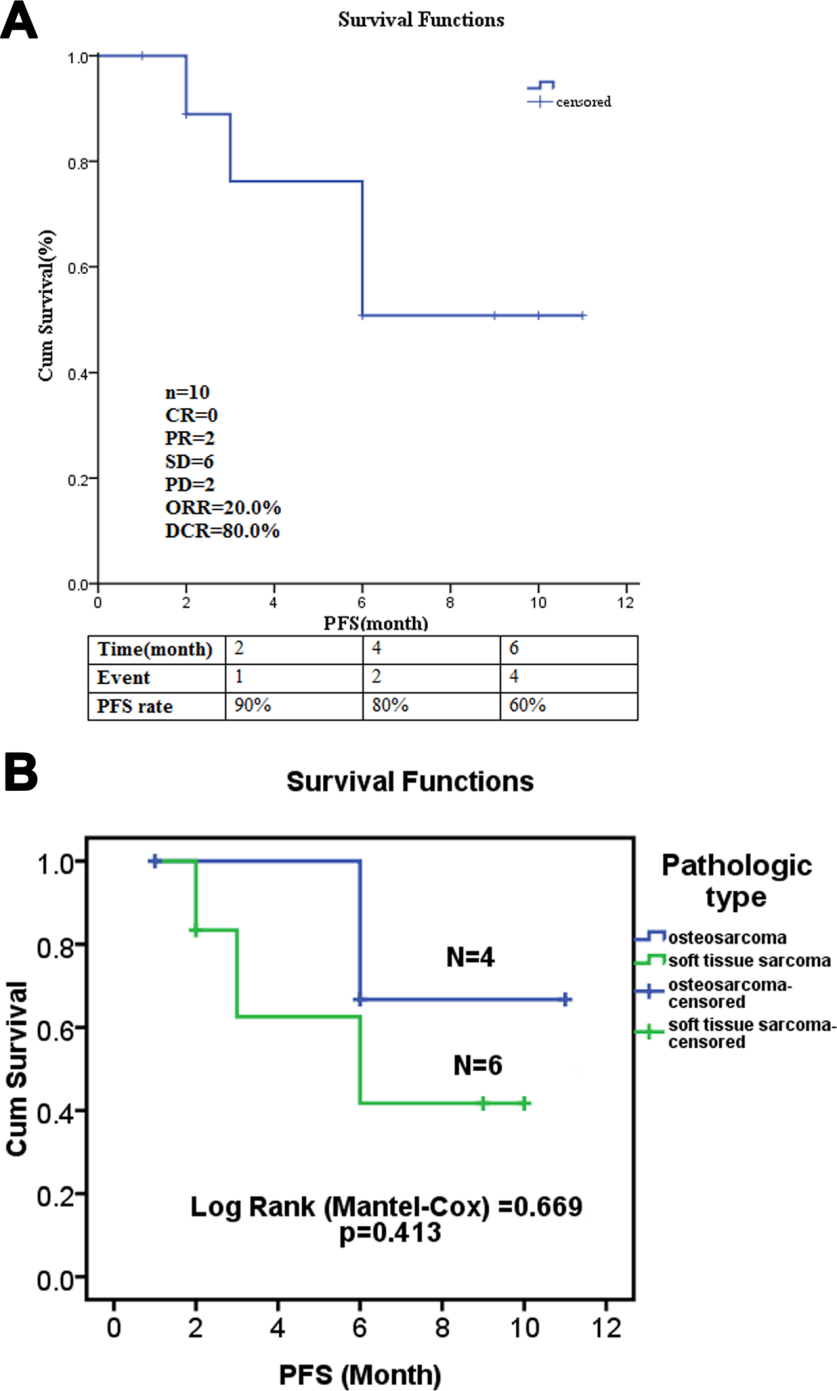
The efficacy evaluation of Apatinib in stage IV sarcoma patients **A**: PFS curve and the clinical response rates of Apatinib therapy. **B**: Different benefit of sarcoma patients with osteosarcoma and soft tissue sarcoma.

**Figure 3:**
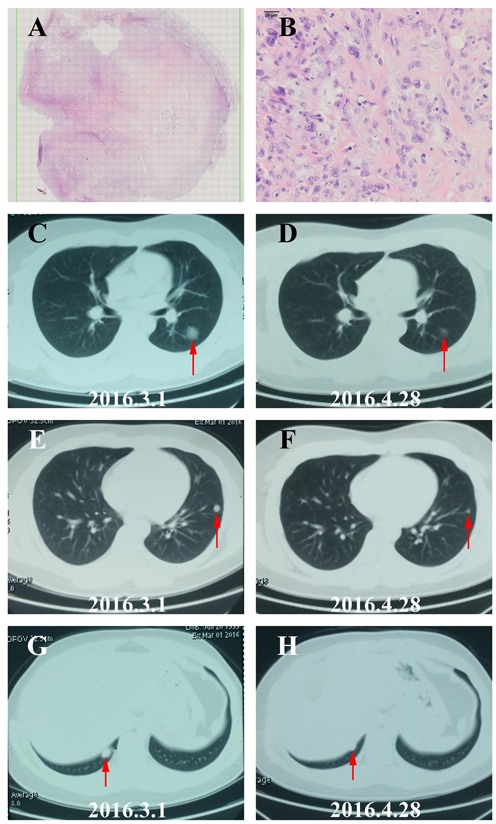
The image and pathological data of one PR patient with lung metastatic osteosarcoma **A**. HE staining of the biopsy tissue from the Lung metastatic lesion (10×). **B**. HE staining of the Lung metastatic lesion demonstrated typical histopathological features of osteosarcoma (40×). **C**.-**H**. The repeated Chest CT showed the result of partial response (PR) with the volumes of lung metastases tumor reducing evidently in 2 months.

**Figure 4:**
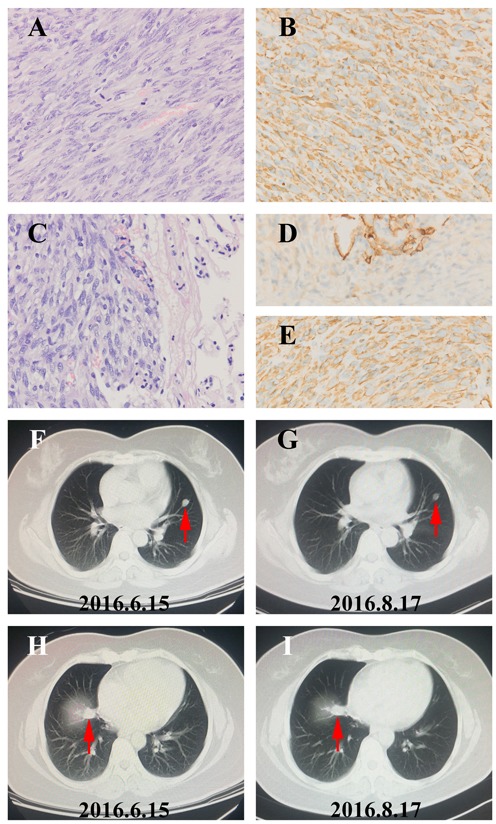
The image and pathological data of one SD patient with lung metastatic soft tissue sarcoma **A**. HE staining of the primary lesion in the hip region showed fibrosarcoma phenotype (40×). **B**. Vimentin protein expression in hip fibrosarcoma (40×). **C**. HE staining of the Lung metastatic lesion after surgery demonstrated typical metastatic fibrosarcoma (40×). **D**. cytokeratin (CK) expression in normal Lung tissue (40×). **E**. Vimentin protein expression in lung metastatic fibrosarcoma (40×). **F**.-**I**. The repeated Chest CT showed the result of stable disease (SD) with the volumes of lung metastases tumor reducing in 2 months.

Ten patients received at least 1 full cycle of Apatinib treatment were eligible for efficacy analysis and 8 patients benefited from the Apatinib therapy (Table 1, Figure 2). The imaging and pathological data of 2 typical cases (1 case with osteosarcoma and another one with STS) were shown as follows. One 16-year-age male patient was first diagnosed to have femur osteosarcoma in August 2014. He was treated by adjuvant chemotherapy, wide resection of tumor, and postoperative chemotherapy with the MTX+DDP+ADM and MTX+IFO+DDP+ADM. After eighth cycles, the CT review found several lung nodes. CT guided biopsy confirmed diagnose of metastatic osteosarcoma in lung (Figure 3A-3B). The patient was treated with 2 cycles of chemotherapy including TXT and GEM. CT scan of the efficacy evaluation concluded progression disease (PD). Then the patient was treated with Apatinib at a 500mg dosage. After taking Apatinib for two cycles, CT review showed significant reduction of multiple metastatic lesions (Figure 3C-3H) and this patient was considered to have partial response (PR) according to Response Evaluation Criteria in Solid Tumors (RECIST) [21]. The patient is currently taking Apatinib with no progression. The second patient is a 36 year old female who was diagnosed to have high grade fibrosarcoma in the hip region in July 2012 (Figure 4A-4B). She was treated with wide surgical resection. In June 2015 the routine CT scan found a nodule in her right lower lung. The patient was treated with right lower lung resection and the postoperative pathological diagnosis was metastatic fibrosarcoma (Figure 4C-4E). After 2 cycles of postoperative chemotherapy regimen with ADM+IFO, the CT scan found multiple nodules in the lungs. The patient began Apatinib regimen with a dosage of 500mg. The first and second monthly CT scan showed a stable disease (SD) (Figure 4F-4I) according to RECIST and she continues to be in stable status 12 months after treatment.

After reviewing all the clinical data of these 10 sarcoma patients, we found the median progress free survival time (PFS) was 8.84 month. The PFS rate was 90% at the second month and 80% at fourth month. The PFS rate decreased to 60% at the sixth month and became stable after that (Figure 2A). In total, there were 2 cases evaluated as PR (20%, 2/10). Six patients (60.0%, 6/10) had SD status till the last follow-up date. Two patients were evaluated as PD and one patient died because of the disease progression (Table 1). The objective response rate (ORR) was 20.0% (2/10) and the disease control rate (DCR) was 80.0% (8/10) (Figure 2A). Metastatic osteosarcoma patients appeared to benefit more than metastatic STS patients although the difference was not statistically significant (Figure 2B). Gender and the previous local disease treatments such as radiotherapy or different surgery types demonstrated no significant effect on the Apatinib effectiveness.

### Toxicity

Toxicities encountered in the study were exhibited in Table 2. Most adverse reactions were mild and easily controlled (grade 1 to 2). Overall, the grade one adverse reactions accounted for 42.9% of the total adverse events. Grade two adverse reactions accounted for 42.9% and 14.2% for grade three, while no grade four was observed in the trial. A total of three patients were treated with a reduction in the dose of the Apatinib during the course of treatment for non-hematologic toxicities. These adverse events, such as Hand-foot syndrome and proteinuria, were quickly reduced and recovered after a dose reduction. So it is critical to detect the toxicity of the drug and adjust the dosage (from 500 mg to 375 mg or 250 mg) of the drug in time. The most frequently observed treatment-related adverse events of grade 3 were as follows: hypertension (18.7%), hand-foot syndrome (12.5%), and proteinuria (6.3%). The most common treatment-related adverse events of all levels were as follows: hand-foot syndrome (43.8%), proteinuria (43.8%), hypertension (31.3%), pain (25.0%), neutropenia (18.8%), bilirubin increased (18.8%), transaminase increased (12.5%), fatigue (12.5%), mucositis (12.5%) and thrombocytopenia (12.5%) (Table 2). There was no drug-related SAEs occurred in this study.

## DISCUSSION

With the in-depth understanding in the pathological mechanisms of tumor, cancer therapy has now entered the era of molecular targeted therapy. In 1971, Folkman firstly proposed that “tumor growth is dependent on the formation of new blood vessels” [22] laying out a new theoretical basis for the control of tumor growth. Over the years, it has been an important research direction in the field of oncology to inhibit the growth of new blood vessels in order to control tumor. Extensive investigations have confirmed that tumor growth and metastasis depend on angiogenesis, and multiple angiogenic factors such as vascular endothelial growth factor (VEGF), bFGF, TGF-β1, MMP, cyclooxygenase-2 (COX-2), CD34, and c-KIT are associated with the prognosis of sarcoma [23]. The expression of VEGF in sarcoma is closely related to the early recurrence, metastasis and the prognosis of the tumor [24].

Apatinib is a oral VEGFR2 inhibitor which could damage the function of human umbilical vein endothelial cells, including proliferation, migration and tube formation [18, 25]. It also breaks the germination of rat aortic rings and inhibits the growth of xenografts, either alone or combining with chemotherapeutic agents [25]. It also could target to side population cells and ABCB1-overexpressing leukemia cells to enhance the efficacy of chemotherapeutic drugs [26].

Due to the high heterogeneity of pathological subtypes of sarcoma, the sensitivity to chemotherapy is variable. Overall, the metastatic sarcoma has a low 5 year survival rate [27]. Thus, new therapeutic strategies for sarcoma are needed. Hereby, we report the first clinical study of Apatinib in stage IV sarcomas to evaluate its efficacy and safety. Our analysis revealed 2 PR and 6 SD patients according to RECIST criteria. The ORR is 20.0% and the DCR is 80.0%. For the long term benefit, the median PFS is 8.84 months and the PFS rate after Apatinib administration was 60% at the sixth month and became stable after that. These data suggest that sarcoma patient could acquire longer benefit from Apatinib treatment. These results are encouraging for the efficacy and seem better than or at least comparable with what was reported in previous studies involved single-agent angiogenesis inhibitors, such as Pazopanib. In a phase 3 trial of Pazopanib reported by Heudel P et al., 246 metastatic soft-tissue sarcoma patients were enrolled, 14 patients achieved PR, 164 patients SD, 57 patients PD, ORR was 6.0% (14/246), DCR was 72.4% (178/246) [27].

Regarding to the safety of Apatinib treatment, the most frequently observed AEs are hand-foot syndrome, proteinuria and hypertension, which are consistent with those reported in gastric cancer and triple negative breast cancer studies [28, 29]. Hypertension can also be controlled by antihypertensive drugs (such as amlodipine, valsartan and so on) in addition to dose disruption or reduction. Hematologic toxicities including neutropenia and thrombocytopenia during treatment does not need to suspend the drug or dose reduction to control because they are mild or moderate.

In summary, our study provides supporting evidence that Apatinib exhibits objective efficacy in stage IV sarcomas with manageable toxicity. Therefore, random controlled trials based on these data are warranted to further evaluate the Apatinib activity in advanced sarcomas.

## MATERIALS AND METHODS

### Patients

The information of 16 patients with stage IV sarcomas who failed in prior chemotherapy treated in Tianjin Medical University Cancer Hospital were collected. The patients suffered from disease progression after first line chemotherapy. All these patients had at least one extracranial measurable site of disease. The initial dose of Apatinib was 500 mg / day and the dose should reduced from 500 mg to 375 mg or 250 mg if there is an intolerable side effect. Application of Apatinib was in accordance with the Declaration of Helsinki and this work was approved by the Ethics Committees of Tianjin Medical University Cancer Hospital. All patients volunteered to participate in this trial and with written informed consent.

### Efficacy and safety assessments

This study intended to find out the efficacy and safety of Apatinib in the stage IV sarcoma patients treated in our sarcoma center. The Clinical benefit response (CBR) was according to RECIST [21]. Complete response (CR) means the tumor completely disappeared more than 1 month. Partial response (PR) means the tumor was reduced by at least 30% for at least 4 weeks. Stable disease (SD) means he sum of the maximum diameter of the target lesion reduced to less than PR, or increased to less than progression disease (PD). PD means that the maximum diameter of the target lesion increases by at least 20%, or new lesions occur. (DCR) = (CR+PR+SD) / total number of cases × 100%, and the objective response rate (ORR) = (CR+PR) / total number of cases × 100%. The disease control was evaluated by PFS, which was defined to be the duration from day of registration forward until progression or death. Assessment of AEs was determined by the Common Terminology Criteria (version 3.0) for AEs. [30].

### Statistical analyses

The survival and safety analysis was performed for patients received at least one dose of Apatinib. Life table and Kaplan-Meier survival curves were used for PFS estimation. Data analyses was performed by applying the SPSS 20.0 software.
